# Supplementary material for: Improved Survival Outcomes with Surgical Resection Compared to Ablative Therapy in Early-Stage HCC: A Large, Real-World, Propensity-Matched, Multi-Centre, Australian Cohort Study
Source: Cancers (Basel). 2023 Dec 7;15(24):5741. doi: 10.3390/cancers15245741 (PMC10742146; doi:10.3390/cancers15245741)
Supplement: Supplementary file 1 [file cancers-15-05741-s001.zip › cancers-2723199-supplementary.pptx]

## Slide 1
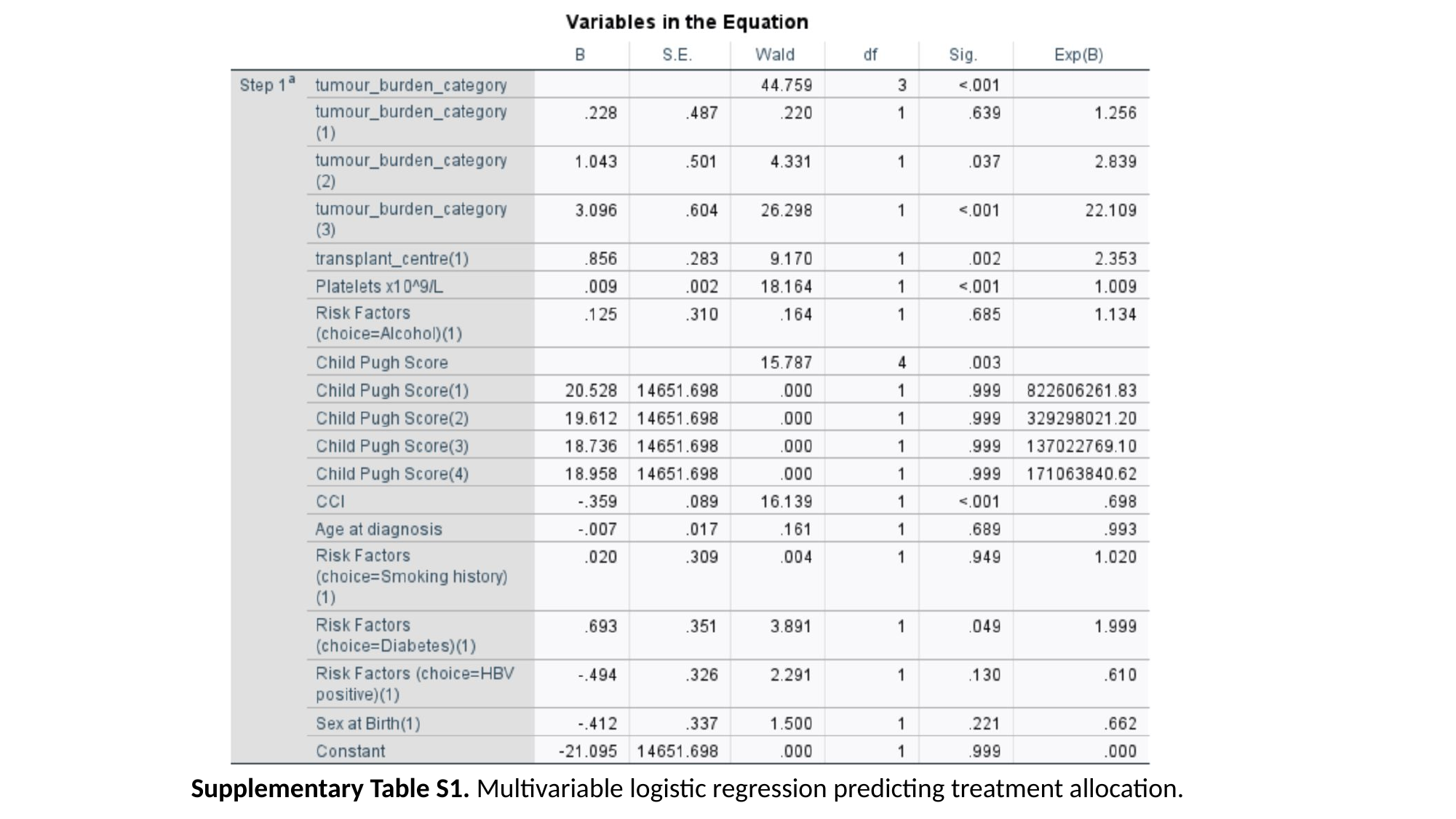

Supplementary Table S1. Multivariable logistic regression predicting treatment allocation.

## Slide 2
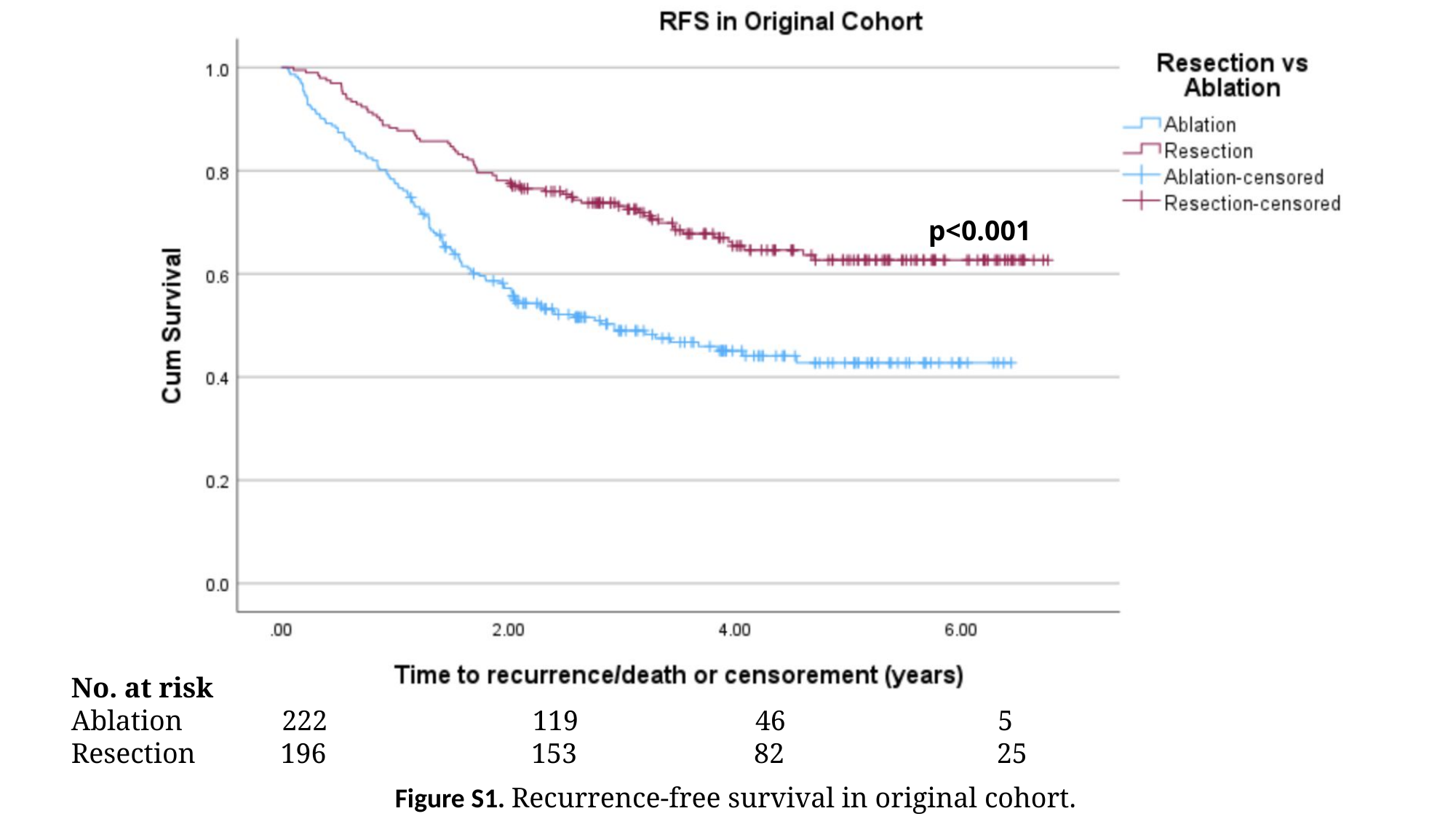

p<0.001
No. at risk
Ablation 222 119 46 5
Resection 196 153 82 25
Figure S1. Recurrence-free survival in original cohort.

## Slide 3
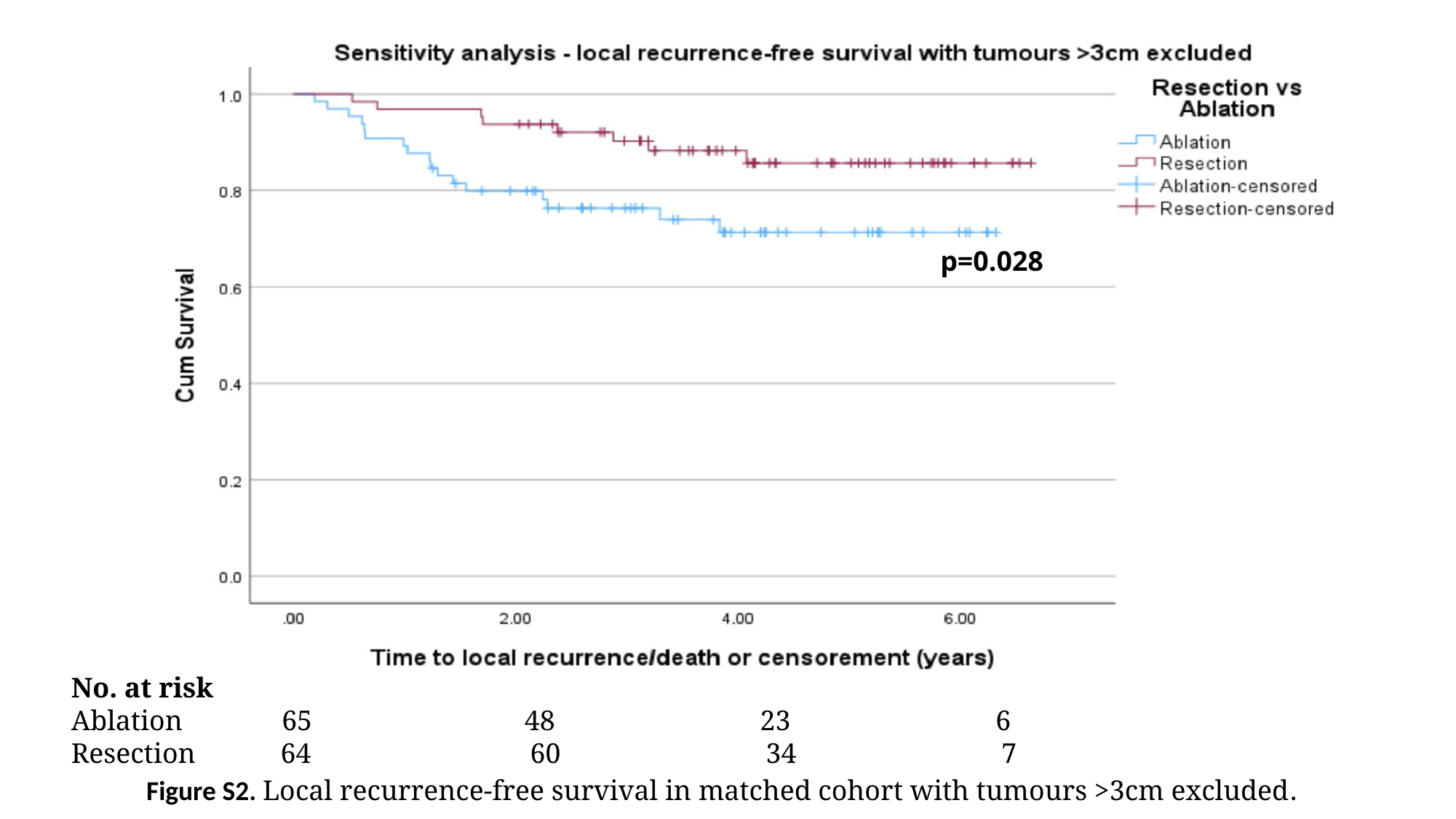

p=0.028
No. at risk
Ablation 65 48 23 6
Resection 64 60 34 7
Figure S2. Local recurrence-free survival in matched cohort with tumours >3cm excluded.

## Slide 4
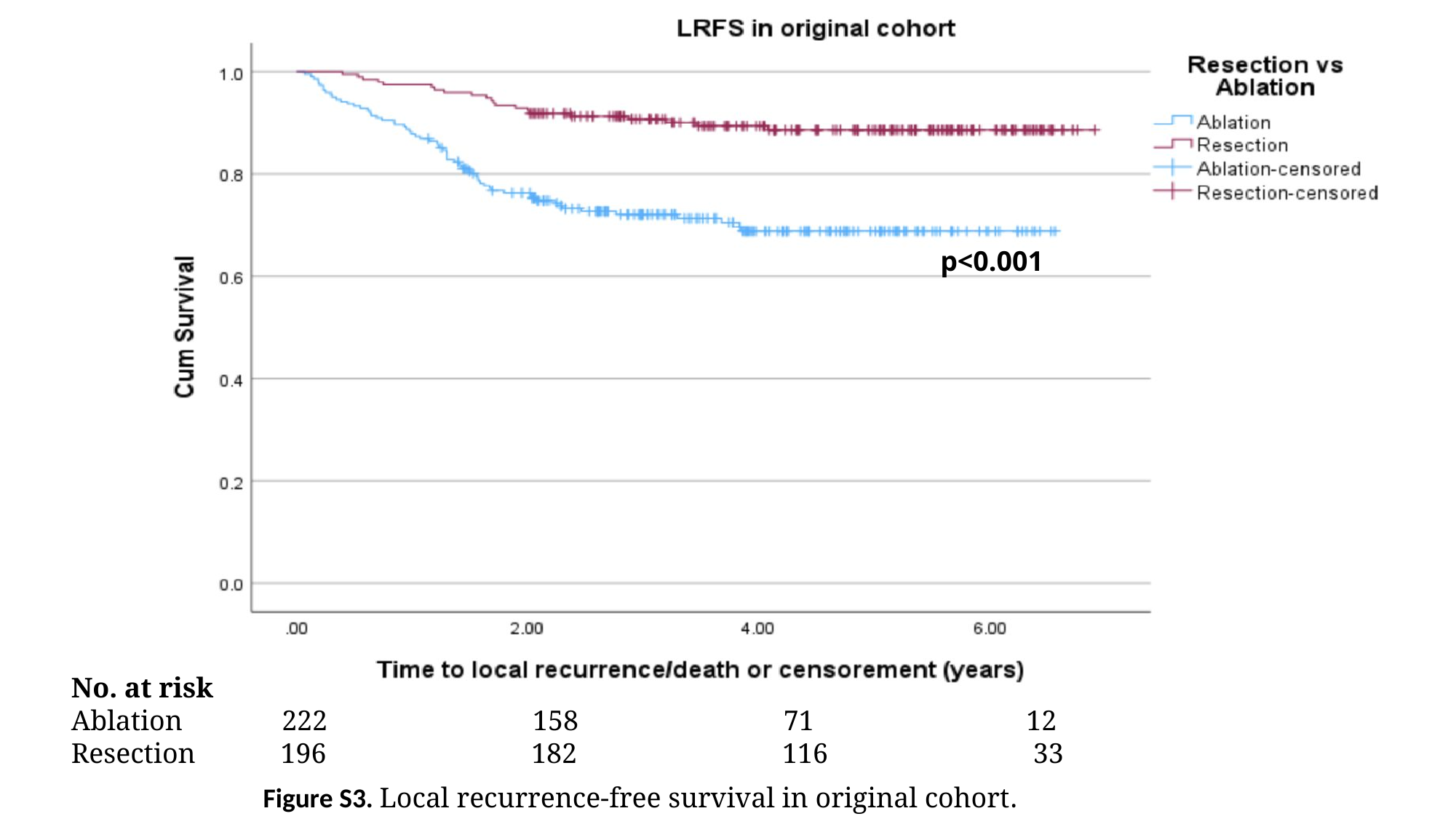

p<0.001
No. at risk
Ablation 222 158 71 12
Resection 196 182 116 33
Figure S3. Local recurrence-free survival in original cohort.

## Slide 5
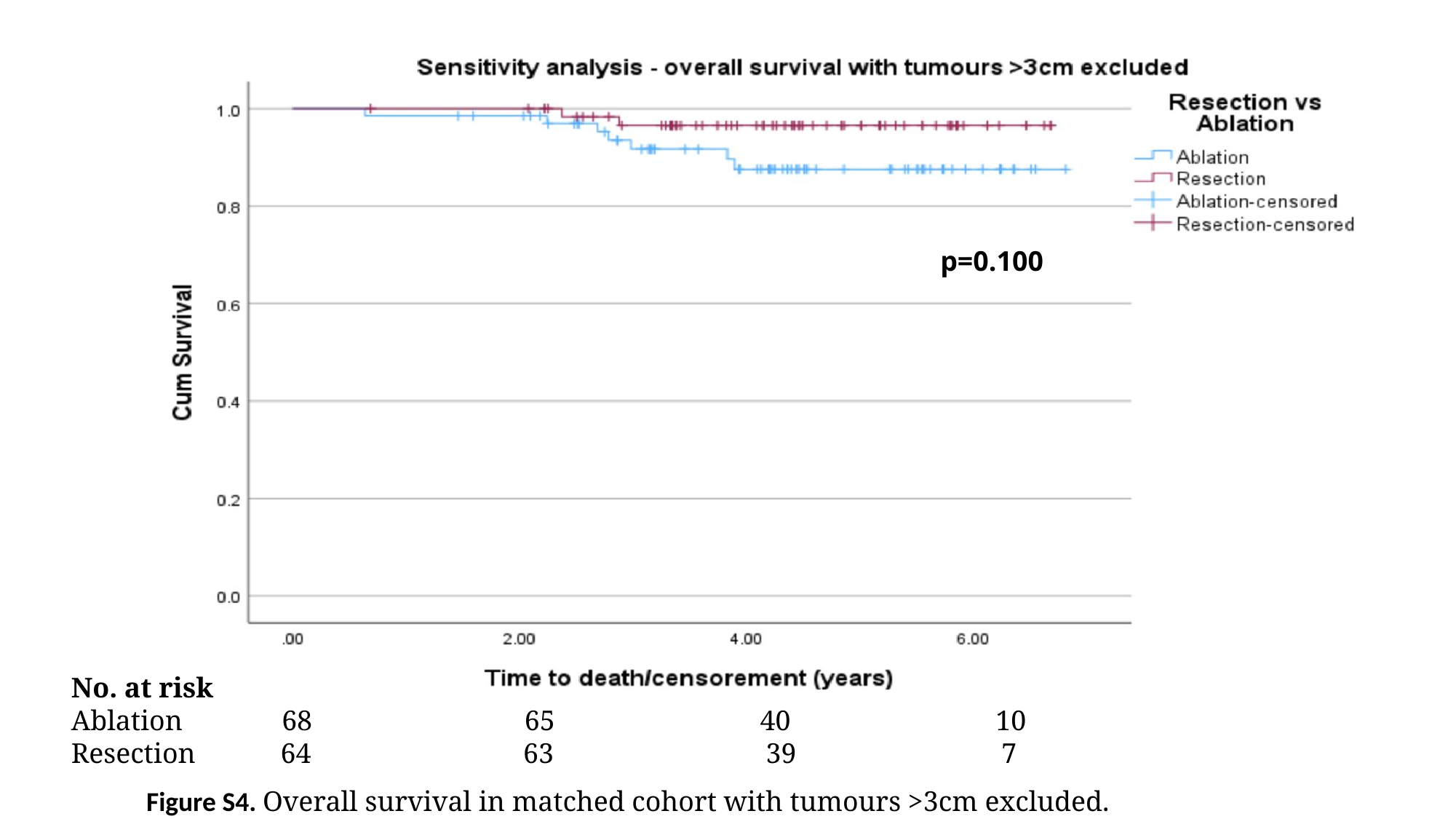

p=0.100
No. at risk
Ablation 68 65 40 10
Resection 64 63 39 7
Figure S4. Overall survival in matched cohort with tumours >3cm excluded.

## Slide 6
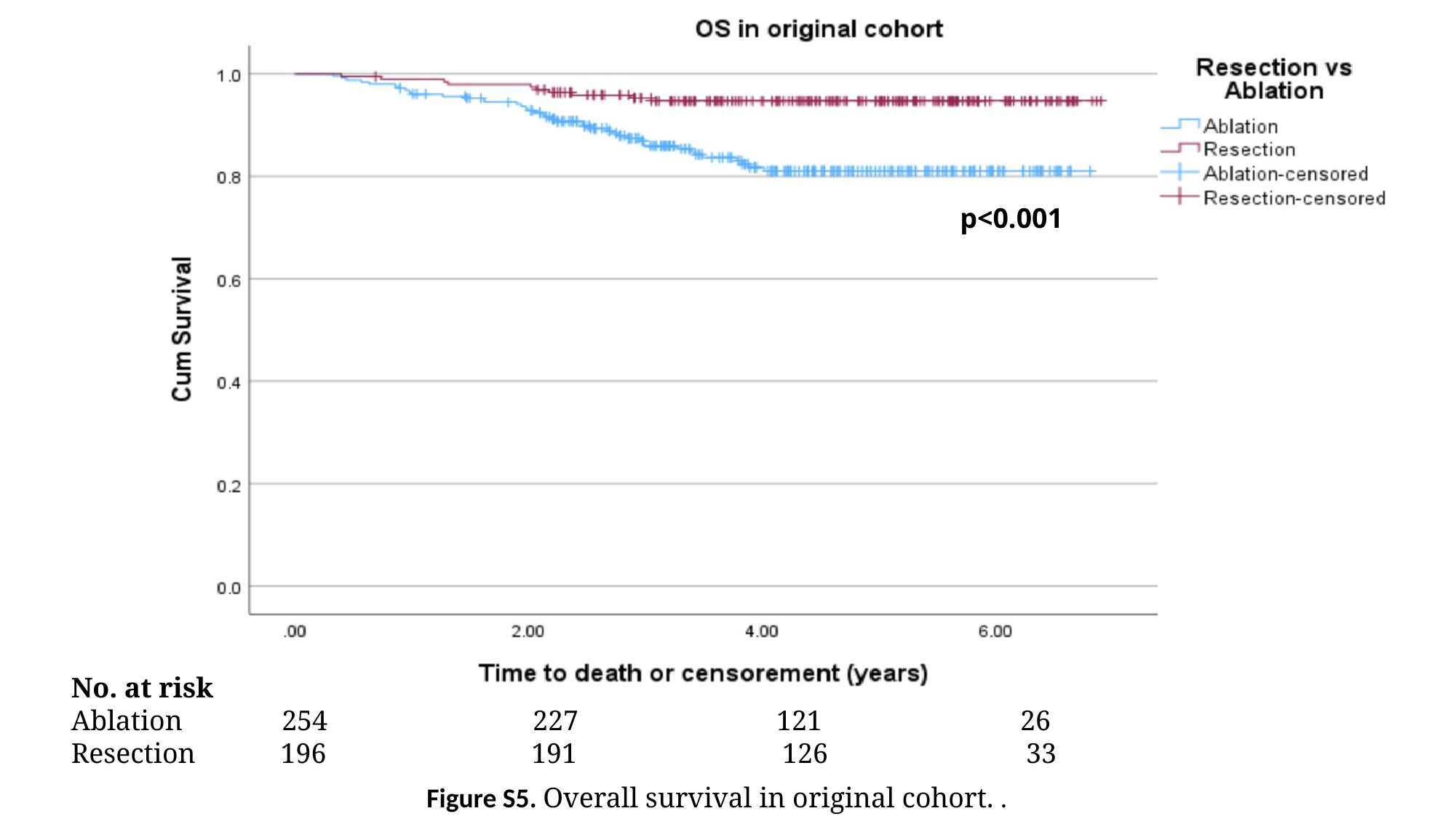

p<0.001
No. at risk
Ablation 254 227 121 26
Resection 196 191 126 33
Figure S5. Overall survival in original cohort. .
